# Christmas at the Hospitals

**Published:** 1888-01-14

**Authors:** 


### CHRISTMAS AT THE HOSPITALS.

(BY OUR COMMISSIONER AND CORRESPONDENTS.)

### AT THE SEAMEN'S HOSPITAL, GREENWICH.

CHRISTMAS DAY falling on a Sunday, the sailors in this hospital (late the *Dreadnought*) had their Christmas dinner on the following day at half-past twelve o'clock. As usual at this charity, the greater number of the nationalities of the world were represented. Scandinavians, Negroes, Icelanders, Chinamen, Persians, Lascars, and natives of every European country, sat down to eat their Christmas dinner together, and those who were too ill to leave their beds had their Christmas fare taken to them—twenty-eight nationalities in all being represented. By a special fund raised for the purpose turkeys were provided for all hands, and there were also plenty of plum-puddings, oranges, and other Christmas fare, which greatly delighted, not only our own British tars, but also those who are strange to this English custom. While the sailors were at dinner many ladies and gentlemen went round the wards, and visited the various dining-halls. Among these were: F. Cleeve, Esq., R.N., C.B. (chairman of the committee of management of the Seamen's Hospital Society), Admiral Sir Thomas Brandreth, K.C.B. (president of the Royal Naval College), and the Rev. Brooke Lambert, M.A., B.C.L. (vicar of Greenwich)). After dinner pipes and tobacco were handed round, and everyone made the best of Christmas "in dry dock." On Friday evening a dramatic and musical entertainment was given in the reading-room. The piece chosen, which was appropriately entitled "Fish Out of Water," was acted by the officers of the hospital, and elicited roars of laughter from a very appreciative audience. The concert with which the entertainment opened was most favourably received, Mr. Charles West and Mr. P. A. Davis (of the Blackheath Dramatic Club) receiving rapturous applause, as did also the other singers, many of whom were old friends of the hospital. On this occasion also many guests were present, among whom was Mr. Henry C. Burdett, a member of the committee, who addressed a few words to the audience at the close of the entertainment. Several shipowners too were present, and it is gratifying to see these gentlemen who benefit by the labours and exertions of sailors, taking so deep an interest in this hospital, which is always open for the treatment of sailors suffering from accident or sickness.

### THE OPHTHALMIC HOSPITAL, MOORFIELDS.

DISEASES of the eye involve a method of treatment which though decorative in a certain way, is not altogether favourable to personal beauty; and perhaps the ladies and gentlemen who assisted at the concert given to the nurses and patients at this hospital on Wednesday, the 4th, never before addressed such a motley audience. The collection of bandaged heads on one side, was balanced by the white caps and aprons of the nurses, and the gay dresses of the guests invited by the hospital officials. All the patients, however, down even to baby Teddy, the three-year-old pet of the institution—suffering, alas! from cataract in both eyes—seemed to enjoy hearing the music. Romberg's Toy Symphony gave special delight, which was not marred by a certain orginality of behaviour on the part of some of the toys; and the admirable recitations of Mr. de Cordova—Calverley's "Gemini and Virgo" and a vigorous ballad entitled "A Fireman's Story"—won both close attention and vigorous applause. Miss Patten showed herself to be possessed of a delightful soprano voice and a finished method; and Mr. Homan's violin playing was remarkably good, his double-stopping being especially noteworthy. There was also some good part-singing; two songs by the Rev. A. W. Clamphett, recitations by Mrs. Burnett Morris, and four songs, which showed wonderful ease in the execution of very elaborate and florid music, by Madame Pauline Rita. All these were received in a fashion that should make the performers ready to come again to brighten another Christmastide for such appreciative listeners. The whole arrangements reflected the greatest credit upon Mrs. Peel, the Matron, and Mr. Robert J. Newstead, the energetic and courteous Secretary.

### THE OLDHAM INFIRMARY.

IF any of the Infirmary inmates were asked this morning what kind of a Christmas they had had, the reply would assuredly be, "A happy one." So much generosity has been shown by members of the public, both by those who have and those who have not hitherto taken a warm interest in the institution; so much thoughtfulness has been displayed in the attention given to the comfort and enjoyment of the patients by the nurses and others, with Miss Thompson, the matron, at their head, that Christmas, 1887, must live as a bright memory in the subjects of their kindliness. The main celebration was on Monday last. The various wards of the institution were decorated tastefully with holly, some of which was sent by Mr. S. Taylor, J.P., and some from the public park. The wards were clean and tidy, and everything was as bright as a new pin—a pattern of neatness. Nor was the appearance of the patients less pleasing. That they had not been forgotten the following list of gifts will show: From the Mayor, apples and oranges, and Christmas cards; from the Mayoress, scarlet jackets; the Misses Rountree, Werneth Hall Road, a Christmas tree; Miss Suthers, Christmas pillow letters; Mrs. Eli Spencer, Pimlico, Christmas cards, periodicals, and flowers; the Editor of *Little Folks*, Christmas cards, periodicals, children's aprons, etc.; Miss Hoyle, Manchester Street, scarlet flannel jackets; Mrs. Potter, High Street, children's slippers, dolls, and books; Mrs. Jos. Wood, Werneth, basket of grapes; same from Dr. and Mrs. Platt; Miss Annette Clegg, Crompton, children's albums and scrap-books; Mr. James Taylor, Queen's Park, apples, oranges, chocolates; Miss Newton, Greenacres, dolls, scrap-books, etc.; and other presents, including gifts from kindly-disposed tradespeople, such as Messrs. Entwistle, Ogden, Holt, and Harrog. The Christmas dinner included turkey and plum-pudding, without which seasonable viands no feast on such an occasion could be complete. They were all provided with a plentiful dessert, which was partaken of in the respective wards. The day was ended with a concert, which took place in the lower men's ward, when all the in-patients, to the number of sixty (twenty-eight men and boys, and thirty-two of the other sex), were present.

### CLAYTON HOSPITAL, WAKEFIELD.

THIS hospital was gaily decorated with flags and evergreens in honour of Christmas, and the matron, Miss Sidebottom, and her assistant, Miss Wilkinson, did all they could to make the patients forget that they were away from home and in an abode of pain. There was a large Christmas-tree, and a dinner of goose and plum-pudding was provided for those who could partake of such fare. A suitable dessert was given by Mr. J. Binks, the hon. secretary of the hospital committee.

### PRINCESS ALICE MEMORIAL HOSPITAL, EASTBOURNE.

ON Wednesday, the 28th ult., a tea and entertainment were given to such of the inmates of this hospital as were able to attend. An amusing series of magic lantern views was exhibited by Mr. C. W. Anderson, the stories illustrated being narrated by Mr. H. D. Farrell. The lantern and views were kindly lent by Dr. H. S. Gabbett. Subsequently gifts were distributed from a beautifully-decorated Christmas-tree, which was loaded with toys, fruit, bon-bons, &c. Great credit is due to the matron, Miss Napper, for her very successful organization of a most happy gathering.

